# The cytochrome oxidase defect in ISC‐depleted yeast is caused by impaired iron–sulfur cluster maturation of the mitoribosome assembly factor Rsm22

**DOI:** 10.1002/1873-3468.70129

**Published:** 2025-08-06

**Authors:** Ulrich Mühlenhoff, Dominik Trauth, Weronika Śliwińska, Linda Boss, Roland Lill

**Affiliations:** ^1^ Institut für Zytobiologie im Zentrum SYNMIKRO Philipps‐Universität Marburg Germany; ^2^ Department of Bioenergetics, Faculty of Biology, Institute of Molecular Biology and Biotechnology Adam Mickiewicz University Poznań Poland

**Keywords:** biogenesis, cytochromes, iron–sulfur protein, mitochondrial DNA, mitochondrial ribosomes, respiratory chain complexes, translation

## Abstract

Mitochondria contain the bacteria‐inherited iron–sulfur cluster assembly (ISC) machinery to generate cellular iron–sulfur (Fe/S) proteins. Mutations in human *ISC* genes cause severe disorders with a broad clinical spectrum and are associated with strong defects in mitochondrial Fe/S proteins, including respiratory complexes I–III. For unknown reasons, complex IV (aka cytochrome *c* oxidase), a non‐Fe/S, heme‐containing enzyme, is severely affected. Using yeast as a model, we show that depletion of Rsm22, the counterpart of the human mitoribosome assembly factor METTL17, phenocopies the defects observed upon impairing late‐acting ISC proteins, that is, diminished activities of mitoribosomal translation and respiratory complexes III and IV. Rsm22 binds Fe/S clusters *in vivo*, thereby satisfactorily explaining the defect of respiratory complex IV in ISC‐deficient cells, because this complex contains three mitochondrial DNA‐encoded subunits.

**Impact statement**

Defects in mitochondrial Fe/S protein biogenesis also impact respiratory complex IV (COX), even though it lacks Fe/S clusters. Here, we show that the mitoribosome assembly factor Rsm22 binds Fe/S clusters in vivo. Rsm22 maturation defects impair mitoribosomal protein translation including COX subunits, explaining the COX defects in Fe/S cluster‐deficient cells.

## Graphical abstract

The yeast mitoribosome assembly factor Rsm22 contains a [4Fe‐4S] cluster that is matured by the mitochondrial iron–sulfur cluster assembly (ISC) machinery. Defects in ISC components result in impaired mitochondrial protein synthesis due to a mitoribosome assembly defect. This causes a deficiency of respiratory complexes III and IV, even though the latter does not contain Fe/S but rather heme cofactors.

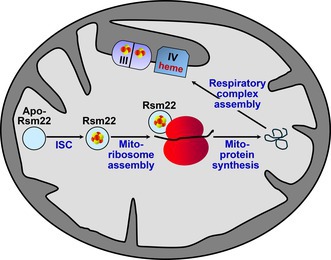

## Abbreviations

**COX**, cytochrome *c* oxidase

**Fe/S**, iron–sulfur

**ISC**, iron–sulfur cluster assembly

**mtSSU**, mitoribosomal small subunit

**SDH**, succinate dehydrogenase

Mitochondria participate in numerous biochemical pathways such as protein translation, oxidative phosphorylation (OxPhos), fatty acid degradation, and the synthesis of cofactors such as iron–sulfur (Fe/S) clusters, heme, and lipoic acid [1, 2, 3, 4, 5, 6, 7]. Many of these biochemical processes are intimately interconnected, with the consequence that a defect in one pathway indirectly affects other processes. The underlying biochemical mechanisms behind these connections are often difficult to unravel. One prominent example is the severe impact of mitochondrial Fe/S protein assembly defects on the respiratory complex IV, aka cytochrome *c* oxidase (COX), even though this multisubunit enzyme contains only heme but no Fe/S cofactors [8, 9, 10]. This so far unexplained issue was the main incentive for the current work.

The biogenesis of mitochondrial Fe/S proteins by the bacteria‐derived iron–sulfur cluster assembly (ISC) system occurs in four consecutive steps and is conserved from yeast to man (reviewed in, e.g., [5, 11, 12, 13, 14]). First, a [2Fe‐2S] cluster is formed *de novo* on the scaffold protein Isu1 (yeast nomenclature) [15, 16]. Initially, sulfur is released from free cysteine by the cysteine desulfurase complex Nfs1‐Isd11‐Acp1. The resulting persulfide sulfur is transferred from Nfs1 to the Fe‐binding Isu1, a reaction stimulated by frataxin Yfh1 [15, 17, 18, 19, 20]. After reduction of the Isu1‐bound persulfide to sulfide by the [2Fe‐2S] ferredoxin Yah1 [15, 19, 21], [2Fe‐2S] cluster synthesis is facilitated by dimerization of two [1Fe‐1S]‐containing Isu1, induced by the conserved N‐terminal tyrosine of Isu1 [22]. Second, the [2Fe‐2S] cluster is transferred from Isu1 to the monothiol glutaredoxin Grx5, a reaction assisted by a dedicated Hsp70‐Hsp40 chaperone system [23, 24, 25]. The Grx5‐bound [2Fe‐2S] cluster is then inserted into target apoproteins without further ISC protein assistance [26].

Third, [4Fe‐4S] clusters are synthesized on the mitochondrial A‐type ISC proteins Isa1, Isa2, and Iba57 by Yah1‐mediated reductive fusion of two [2Fe‐2S] clusters after their acquisition from Grx5 [8, 9, 26, 27, 28, 29, 30]. Finally, [4Fe‐4S] target proteins receive their clusters from Isa1‐Isa2‐Iba57, in some cases with the additional involvement of dedicated ISC targeting factors such as Ind1, Nfu1, and Bol1‐Bol3 [31, 32, 33]. Genetic mutations in almost all human ISC factors cause severe, often lethal disorders with a wide clinical spectrum [5, 34, 35, 36]. As mentioned above, apart from associated biochemical defects in well‐known Fe/S proteins such as aconitase, lipoyl synthase, and respiratory complexes I (not present in *Saccharomyces cerevisiae*), II, and III, there is a so far unexplained strong impairment of the non‐Fe/S, heme‐containing complex IV (aka cytochrome *c* oxidase or COX) [8, 9]. Likely, this is not due to a heme synthesis deficiency, because human ferrochelatase holds a [2Fe‐2S] cluster, yet also late‐acting ISC protein depletion causes this defect [9, 37]. In yeast, heme b synthesis does not even involve Fe/S proteins. Conspicuously, the COX defect is particularly strong upon depletion of the late‐acting ISC complex Isa1‐Isa2‐Iba57, but the synthesis of the COX‐specific cytochromes *a/a*_*3*_ only requires the core ISC‐dependent [2Fe‐2S] ferredoxin Yah1, but no [4Fe‐4S] proteins [6, 38]. Moreover, yeast strains deficient in any of the late‐acting ISC proteins lose their mitochondrial DNA (mtDNA) for unknown reasons, similar to many strains carrying gene deletions of mitoribosomal proteins [39].

Here, we investigated the molecular reason for the COX defect upon ISC protein dysfunction, in particular of the late‐acting ISC proteins Isa1, Isa2, and Iba57. We identify the *S. cerevisiae* mitochondrial protein Rsm22, a homolog of the human mitoribosome assembly factor METTL17, as an Fe/S protein *in vivo*, and show that its depletion leads to biochemical consequences that are similar to (late‐acting) ISC protein deficiencies, including severe defects in mitochondrial translation and COX activity.

## Materials and methods

### Yeast strains, growth conditions and yeast vectors

Yeast strains (W303‐1A background; see Table S1; Refs [16, 27, 40, 41, 42, 43, 44, 45, 46, 47]) were cultivated in rich (YP) and synthetic complete (SC) minimal medium supplemented with the required amino acids and 2% (w/v) of indicated carbon sources [48]. Cells carrying *GAL1‐10* or *GAL‐L* promoter exchanges (Table S1) were depleted by cultivation in rich YPD medium for 40 h (Gal‐*IBA57*, Gal‐*ISA12*; Gal‐*Hem15*, Gal‐*NFS1*, Gal‐*MRPS5*, Gal‐*RSM22*, and Gal‐*YAH1* cells) or 64 h (Gal‐*ISA1*, Gal‐*SSQ1* and Gal‐*YFH1*) with intermediate dilutions to OD_600nm_ = 0.1 after 16 and 40 h. Rsm22 was overproduced from vector p424‐TDH3 carrying the coding information of Rsm22 and a C‐terminal HA‐tag.

### Biochemical methods

For *in vivo* ^55^Fe radiolabeling, yeast cells overexpressing Rsm22‐HA were grown for 40 h in iron‐poor medium, and were then radiolabeled with ^55^FeCl_3_ (Perkin‐Elmer) for 2 h [49, 50]. After glass‐bead lysis, the clarified whole‐cell extract was used for affinity precipitation of Rsm22‐HA. The amount of ^55^Fe associated with the affinity beads was quantitated by scintillation counting.

Enzyme activities were measured in clarified whole‐cell extracts obtained by glass‐beads lysis following procedures published in dedicated methods papers [49, 50, 51, 52]. In brief, aconitase (Aco) activity was followed by a coupled assay with isocitrate dehydrogenase (IDH) by reduction of NADP^+^ monitored at 340 nm. For malate dehydrogenase (MDH), NADH oxidation at 340 nm was recorded. Respiratory complex IV (cytochrome c oxidase, COX) was followed by oxidation of reduced cytochrome *c* monitored at 550 nm. Succinate dehydrogenase (SDH/DCPIP assay) was assayed by monitoring the reduction of 2,6‐dichlorophenolindophenol (DCPIP) at 600 nm in the presence of succinate and phenazine methosulfate. Measurement of complex II + III activity (SDH/CytC assay) was performed by monitoring the reduction of cytochrome *c* at 550 nm in the presence of succinate. Enzyme activities for the tested strains were measured in parallel with corresponding wild‐type control strains and normalized to MDH activity (U/mg). Ferrochelatase activities were measured by following the fluorescence emission at 588 nm (excitation at 418 nm) associated with the insertion of Zn^2+^ into protoporphyrin [53]. For reduced‐minus oxidized cytochrome spectra, 1 mg isolated mitochondria was diluted in 1 mL 100 mm Tris–HCl, pH 7.5 containing 0.5% Triton X‐100 and 1 mm potassium hexacyanoferrate III. The clarified lysate was split equally into two cuvettes. One cuvette was reduced with 10 mm sodium dithionite for 2 min, and the visible spectrum between 650 and 500 nm was recorded against the oxidized reference cuvette.

Translation of mtDNA‐encoded mitochondrial proteins was measured by *in vivo* labeling of yeast cells with ^35^S methionine (Hartmann Analytic) [54]. In brief, cells were resuspended in 250 μL SD medium supplemented with 1 mg cycloheximide at OD_600_ = 3. After 10 min, cells were supplemented with 60 μg of all canonical amino acids (except methionine) plus 15 μCi [^35^S]‐methionine and incubated for 20 min at 30 °C. Incorporation of [^35^S]‐methionine into proteins was stopped by the addition of 0.5 mg·mL^−1^ chloramphenicol and 4 mm methionine. Cells were lysed by alkaline lysis; cell extracts were subjected to SDS/PAGE and blotted to nitrocellulose membranes. Autoradiograms were recorded using a Sapphire FL biomolecular imager (Azure Biosystems, Dublin, CA, USA).

Mitochondrial DNA was visualized by staining yeast cells cultivated in minimal SD medium to mid‐log phase (OD_600_ ~ 0.5–1) with 4′,6‐diamidino‐2‐phenylindole (DAPI) [55]. Briefly, yeast cells precultivated in SD medium for 40 h were grown for 4 h in minimal SD medium to mid‐log phase (OD_600_ ~ 0.5–1). Cells from a 1 mL culture were harvested by centrifugation, washed with 1 mL water, resuspended in 1 mL ethanol, and incubated with 0.1 μg·mL^−1^ DAPI for 5 min in the dark. Cells were harvested, washed twice in 1 mL water, and resuspended in 20 μL water. 5 μL of this suspension was mixed with 15 μL Moviol and placed on microscope slides. Fluorescence images were recorded with a DMI600B Microscope (Leica, Wetzlar, Germany) at 100‐fold magnification.

### Miscellaneous standard methods

Basic molecular biological, genetic, biochemical, and immunological methods were performed according to standard technical publications: Manipulation of DNA and PCR [56]; transformation of yeast cells [57]; preparation of yeast mitochondria and cell extracts [58]; immunological techniques [59]. Statistical analyses were carried out with GraphPad Prism 3. Error bars indicate the SEM.

## Results

### COX deficiency develops fast in yeast cells depleted of Isa1 and Isa2

**Fig. 1:**
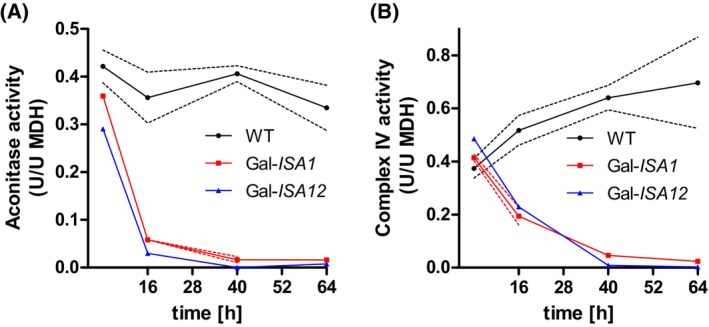
Rapid loss of cytochrome oxidase activity upon depletion of Isa1 and Isa2 in *S. cerevisiae*. Wild‐type (WT), Gal‐*ISA1* and Gal*‐ISA12* cells (W303‐1A background) were initially cultivated in YPGal medium, and then inoculated at OD_600nm_ = 0.1 in rich YPD medium. After 4, 16, 40, and 64 h, enzyme activities of aconitase (A) and complex IV (COX) (B) were determined in cell extracts. At 16‐h and 40‐h time points, cells were transferred into fresh YPD medium. Date are presented relative to the activity of malate dehydrogenase (MDH). Dotted lines indicate the SD (*n* ≥ 4).

The COX activity defect upon depletion of the late‐acting ISC proteins Isa1, Isa2, and Iba57 could be an unspecific, long‐term effect. We therefore compared the rate of activity loss of the non‐Fe/S‐dependent COX enzyme with that of the Fe/S marker protein aconitase in cells depleted of Isa1 and/or Isa2 [45, 60, 61]. We used galactose‐regulatable strains in which the levels of Isa1 (Gal‐*ISA1* cells) and Isa1 plus Isa2 (Gal‐*ISA12*) can be depleted upon cultivation in glucose‐containing, rich growth medium [8]. In both Gal‐*ISA1* and Gal‐*ISA12* cells, the enzymatic activity of aconitase dropped drastically upon Isa protein depletion after cultivation in YPD medium for 16 h, and was almost undetectable after 40 h (Fig. 1A). The activity of COX decreased to 40% of wild‐type levels after 16 h and reached background levels after 40‐h cultivation (Fig. 1B). The only slight delay of COX activity loss compared to aconitase could be due to protein stability differences, and thus, this result pointed more towards a specific, even though somewhat belated requirement of Isa1 and Isa2 in COX maturation. While the results argue against an unspecific long‐term pleiotropic consequence, they do not provide any further clues into the molecular reasons of the COX defect.

### Impaired COX activity in late‐acting ISC protein‐depleted cells is not caused by heme or complex III defects

**Fig. 2:**
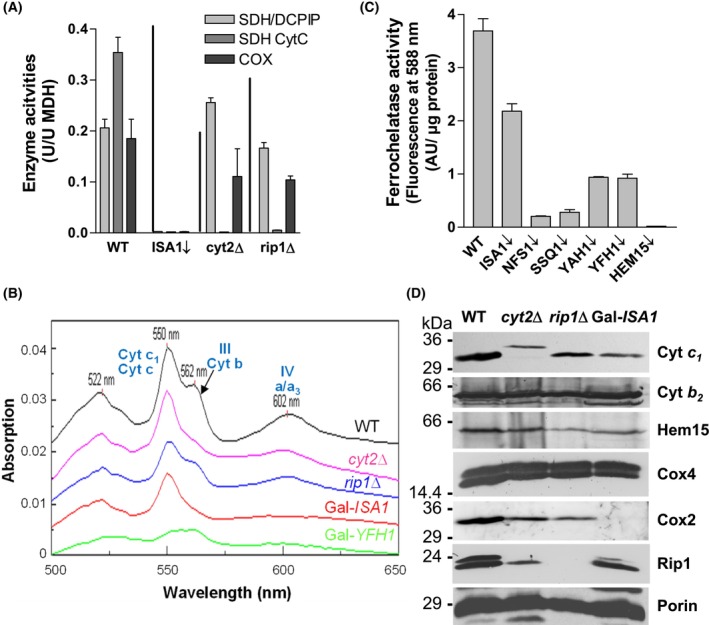
Impaired COX activity upon depletion of Isa1 is not caused by a heme or complex III defect. Mitochondria were isolated from wild‐type (WT), Gal‐*ISA1* (ISA1↓; depleted for 40 h), *cyt2Δ*, and *rip1Δ* cells (the latter two being deletion mutants with a defective respiratory complex III) after cultivation in YPD medium. Mitochondria were analyzed for (A) enzyme activities of respiratory complexes II (SDH (DCPIP)), II and III (SDH (CytC)), and IV (COX) relative to malate dehydrogenase (MDH), or (B) reduced‐minus‐oxidized cytochrome spectra. The peaks of the various cytochrome types are indicated. Depleted Gal‐*YFH1* cells (frataxin) served as a control. The individual spectra were offset for better visualization. (C) Ferrochelatase activities of mitochondria isolated from wild‐type or depleted Gal (↓) yeast strains of the indicated genes were determined by following the fluorescence emission at 588 nm (excitation at 418 nm) associated with the insertion of Zn^2+^ into protoporphyrin IX. (D) Levels of the indicated proteins from mitochondria of part A were determined by Western blotting. Porin served as a loading control. The apparent molecular masses of marker proteins are indicated in kDa. Error bars indicate the SD (*n* ≥ 3).

COX tightly interacts with respiratory complex III (aka cytochrome *bc*_*1*_ complex) as a supercomplex [62, 63]. Since both complexes contain (different) hemes as cofactors, it seems conceivable (but not likely; see Introduction) that the loss of a functional supercomplex III‐IV and/or a heme deficiency may be the molecular reason for the COX defect in late‐acting ISC protein‐depleted cells. In contrast to this hypothesis, yeast cells lacking a functional cytochrome *c*_*1*_ subunit (strain *cyt2*Δ) or the Rieske Fe/S protein (*rip1*Δ), and therefore containing a nonfunctional complex III (Fig. 2A, cytochrome *c* reduction assay), retained COX activity, although at slightly diminished levels (50–60% of wild‐type) (Fig. 2A). As a control, the activity of respiratory complex II (SDH) was similar to wild‐type in these two mutant cells, but was lost completely upon depletion of Isa1, consistent with the presence of [4Fe‐4S] clusters in this complex (Fig. 2A, DCPIP assay).

Cytochrome spectra of mitochondria isolated from *cyt2*Δ or *rip1*Δ cells showed the characteristic peak for heme *a/a*_*3*_ of COX (at 602 nm) (Fig. 2B). On the contrary, in depleted Gal‐*ISA1* cells, this heme *a/a*_*3*_ peak and that of cytochrome *b* of complex III (562 nm) were fully absent. The lack of *a/a*_*3*_‐ and *b*‐type cytochromes in Isa1‐depleted cells does not appear to be related to a heme‐biosynthesis defect. First, mitochondria isolated from Gal‐*ISA1* cells (Isa1‐depleted in YPD) contained active ferrochelatase, although at slightly diminished levels (60% of wild‐type) (Fig. 2C). In contrast, core ISC protein‐depleted cells (Gal‐*NFS1*, Gal‐*SSQ1*, Gal‐*YAH1*, and Gal‐*YFH1* cells) contained rather low levels of ferrochelatase activity. As a control, ferrochelatase‐depleted Gal‐HEM15 cells completely lacked this activity. Second, the cytochrome *c*_*1*_ subunit of complex III was fully processed in Gal‐*ISA1* cells (Fig. 2D). The processing of the sorting sequence of the intermediate form of cytochrome *c*_*1*_ is strictly dependent on the presence of heme and its covalent attachment by the heme lyase enzyme Cyt2 [64, 65, 66] (Fig. 2D). Hence, the presence of fully processed cytochrome *c*_*1*_ in depleted Gal‐*ISA1* cells was another clear indication of the presence of heme. Third, the levels of cytochrome *b*_*2*_, a soluble heme protein of the mitochondrial intermembrane space, were normal (Fig. 2D). Fourth, the UV–Vis spectrum of mitochondria isolated from Gal‐*ISA1* cells (as those from *cyt2*Δ or *rip1*Δ cells) showed a signal for *c*‐type cytochromes (at 550 nm; 40% of wild‐type), indicating that these cells grown under fermentative conditions can generate heme (Fig. 2B). We note, however, that under respiratory growth conditions, where *ISA1* becomes essential for cell viability, Gal‐*ISA1* cells develop a stronger heme deficiency reminiscent of yeast cells with low levels of core ISC factors such as frataxin Yfh1‐depleted Gal‐*YFH1* cells (compare Fig. 2B,C and Ref. [67]). Finally, the formation of heme *a* from heme *b* does not involve [4Fe‐4S] proteins, and rather requires the mitochondrial [2Fe‐2S] ferredoxin (yeast Yah1 or human FDX2), the maturation of which does not involve late‐acting ISC factors [6, 38, 68]. Collectively, these results strongly suggest that the severe COX deficiency in Gal‐*ISA1* cells is not connected to a (potential) malfunction of complex III and hence failed supercomplex III‐IV formation or a heme deficit.

Strikingly, we found that the COX subunit Cox2, which is encoded by the mtDNA, was virtually absent in depleted Gal‐*ISA1* cells, in clear contrast to findings for *cyt2*Δ or *rip1*Δ cells (Fig. 2D). Since the levels of the nucleus‐encoded COX subunit Cox4 were similar in all analyzed cells and since depleted Gal‐*ISA1* cells do not lose mtDNA unlike *isa1*Δ cells [60, 61], this finding may indicate a specific block in mitochondrial transcription and/or translation upon depletion of late‐acting ISC proteins.

### Rsm22 is a mitochondrial Fe/S protein that is matured by the late‐acting ISC system

**Fig. 3:**
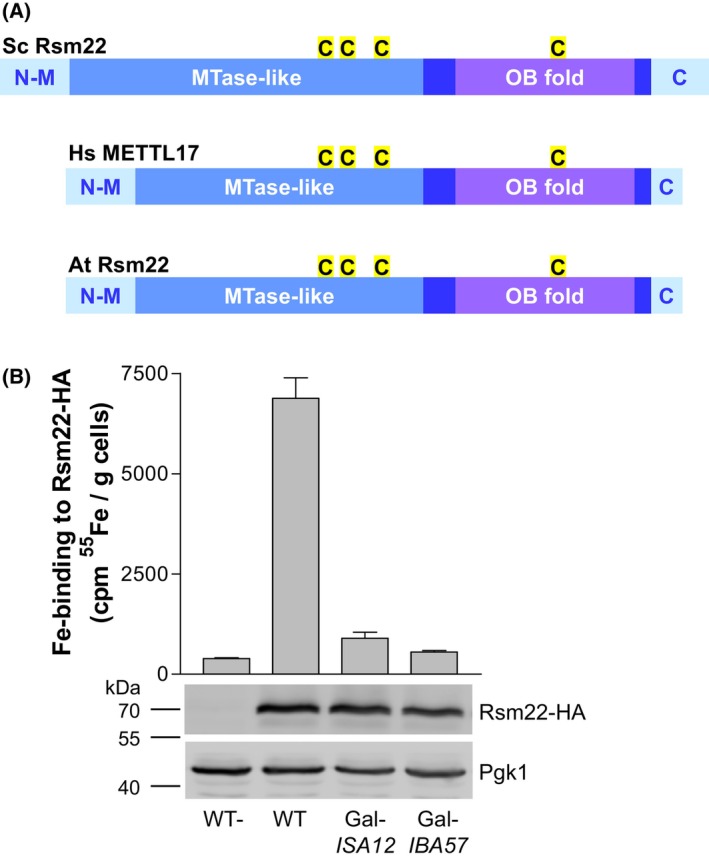
The mitoribosomal assembly factor Rsm22 binds a Fe/S cluster in a late‐acting ISC‐dependent fashion. (A) Cartoon for the domain structure of fungal, vertebrate, and plant members of the Rsm22/METTL17 protein family [73]. Even though the overall sequence similarity is low in eukaryotes, the proteins share a middle methyltransferase‐like (MTase‐like) domain and an oligonucleotide‐binding fold (OB‐fold) within a conserved C‐terminal segment (dark blue). The N‐terminal non‐conserved region contains a mitochondrial targeting sequence (N‐M), while the C termini (blue C) are quite variable. For detailed alignments see Fig. S1A–C. At, *A. thaliana*; Hs, *H. sapiens*; Sc, *S. cerevisiae*. (B) Wild‐type (WT), Gal‐*ISA12*, and Gal*‐IBA57* cells overproducing C‐terminally HA‐tagged Rsm22 from vector p424‐TDH3 were radiolabeled *in vivo* with ^55^Fe in iron‐poor SD minimal medium. Wild‐type cells carrying an empty plasmid (WT‐) served as background control. Before the radiolabeling, Gal‐*ISA12* and Gal*‐IBA57* cells were depleted by cultivation in SD medium for 64 h. Cell extracts were prepared, Rsm22‐HA was immunoprecipitated with α‐HA antibodies, and ^55^Fe associated with the immunobeads was quantified by scintillation counting. Protein levels of Rsm22‐HA in radiolabeled extracts was assessed by immunostaining with α‐HA antibodies (bottom part). The cytosolic protein Pgk1 served as a loading control. Molecular masses of marker proteins are given in kDa. Error bars indicate the SD (*n* ≥ 6).

A recent study identified and characterized the human methyltransferase‐like [4Fe‐4S] protein METTL17, which according to electron cryogenic microscopy (cryo‐EM) is attached to the mitoribosomal small subunit (mtSSU), and was shown to support late stages of mtSSU assembly [69, 70]. The primary sequence of METTL17 is poorly conserved in eukaryotes, yet it exhibits weak similarity to *S. cerevisiae* Rsm22, a predicted SAM‐dependent RNA methyltransferase that was shown to bind to the mtSSU in yeast [71] (Fig. 3A, Fig. S1A–C; Refs [69, 70, 72, 73, 74]). Eukaryotic Rsm22‐METTL17‐like proteins contain a nonconserved N‐terminal region with a mitochondrial targeting sequence, a middle methyltransferase‐like domain, and a fairly well‐conserved C‐terminal domain, which includes an oligonucleotide‐binding (OB) fold followed by three short segments that are unique to different classes of eukaryotes (Fig. 3A, Fig. S1A–C). These three segments tightly interact with each other and a conserved WP dipeptide at the start of the OB fold, as seen in 3D structures of Rsm22 and human METTL17 [69, 70] (Fig. S1D). All proteins contain four conserved cysteine residues that were found to coordinate a [4Fe‐4S] cluster in cryo‐EM structures of yeast and human mitoribosomal particles and in purified human METTL17 [69, 70].

We used an *in vivo* ^55^Fe radiolabeling‐immunoprecipitation assay [50] to explore whether the electron densities observed in the cryo‐EM studies represent an Fe/S cluster. We further examined the ISC dependency of cluster maturation to verify the presence of a [4Fe‐4S] cluster type in Rsm22. To this end, we overproduced Rsm22 with a C‐terminal HA‐tag in wild‐type yeast cells and investigated the incorporation of ^55^Fe into Rsm22‐HA. Cells were first cultivated in iron‐poor minimal SD medium for 16 h, and then were radiolabeled with ^55^Fe for 2 h. Whole‐cell lysates were prepared, Rsm22‐HA was immunoprecipitated with specific anti‐HA antibodies, and the amount of ^55^Fe bound to washed immunobeads was quantified by scintillation counting (Fig. 3B). ^55^Fe immunoprecipitated from wild‐type cells overproducing Rsm22‐HA was 10‐fold higher than the background levels obtained with cells carrying the empty vector. In Gal‐*ISA12* cells depleted of both Isa1 and Isa2 for 40 h, the amount of immunoprecipitated ^55^Fe bound to Rsm22‐HA was close to background levels. A similar strong diminution of Rsm22‐HA‐bound ^55^Fe was obtained upon depletion of Iba57 in Gal‐*IBA57* cells (Fig. 3B). Since the levels of Rsm22‐HA were comparable in all studied cells (Fig. 3B, bottom), the strict Isa1‐Isa2 and Iba57 dependence of ^55^Fe binding to Rsm22‐HA verifies that this protein binds a [4Fe‐4S] rather than [2Fe‐2S] cluster. We therefore hypothesized that the lack of a [4Fe‐4S] cluster in Rsm22 of Isa1‐Isa2‐ or Iba57‐depleted cells may lead to mitoribosomal assembly and subsequent protein translation defects that in consequence may elicit a functional loss of the OxPhos complexes III‐V, because they all contain mtDNA‐encoded subunits (note that *S. cerevisiae* does not contain a complex I).

### Cells depleted of Rsm22, Isa1, or Isa2 display a mitoribosomal translation defect

**Fig. 4:**
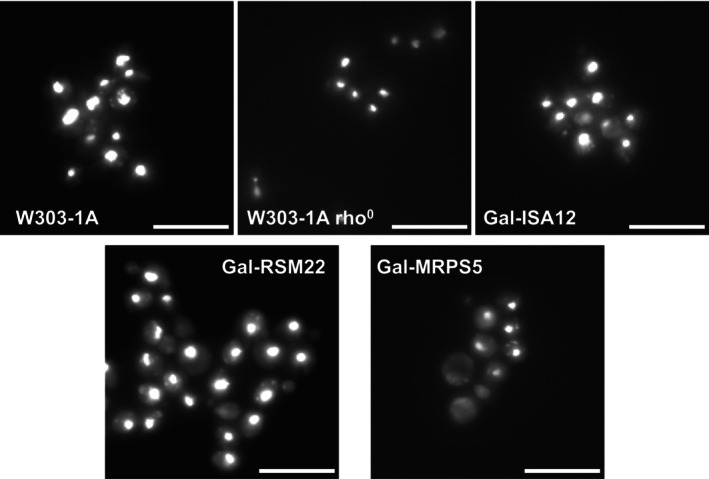
Depleted Gal‐*RSM22*, Gal*‐MRPS5*, and Gal‐*ISA12* cells retain mtDNA. The indicated yeast strains were cultivated for 40 h in SD minimal medium, in order to deplete the respective Gal‐strains to critical levels (Fig. 1; Fig. S2). Cells were cultivated to mid‐log phase in SD medium, stained with DAPI, and analyzed in a DMI6000B fluorescence microscope at 100‐fold magnification (scale bars are 10 μm). W303‐1A and W303‐1A rho^0^ cells served as controls. Due to different staining efficiencies, the exposure times for the different strains vary slightly.

To experimentally verify this hypothesis, we determined the dependence of mitochondrial protein translation on Rsm22 or components of the late‐acting ISC system. To achieve this, we first needed to construct the regulatable Gal‐*RSM22* strain that allowed the repression of *RSM22* upon cultivation on glucose‐containing medium, similar to Gal‐*ISA1* cells. As a control, we generated the strain Gal‐*MRPS5* that can be depleted of Mrps5 (aka uS5m; [75, 76]), a component of the mtSSU with a binding site in the vicinity of Rsm22. As expected, both Gal‐*RSM22* and Gal‐*MRPS5* cells failed to grow on non‐fermentable carbon sources (Fig. S2A,B), similar to rho^0^ cells that lack mtDNA (Fig. S2C). Consistently and in contrast to *rsm22Δ*, *mrps*5*Δ*, *isa1Δ*, *isa2*, and *isa12Δ* deletion cells that have lost their mtDNA [45, 60, 61, 71], the corresponding depleted Gal‐strains retained their mtDNA as documented by staining with fluorescent dye DAPI (Fig. 4).

We further scored for the presence of mtDNA by mating Gal‐*RSM22* and Gal‐*MRPS5* cells depleted for 40 h in YPD with a mtDNA‐devoid rho^0^ tester strain [48]. The resulting diploid cells were capable of growing on agar plates containing the nonfermentable carbon source glycerol (Fig. S2C), and formed similar numbers of colonies as wild‐type cells, whereas rho^0^ cells formed no colonies (Fig. S3). These data clearly demonstrate that Gal‐*RSM22* and Gal‐*MRPS5* cells retained their mtDNA upon protein depletion, thus mimicking the behavior of Gal‐*ISA1*, Gal‐*ISA2*, and Gal‐*IBA57* cells [27, 45, 61].

**Fig. 5:**
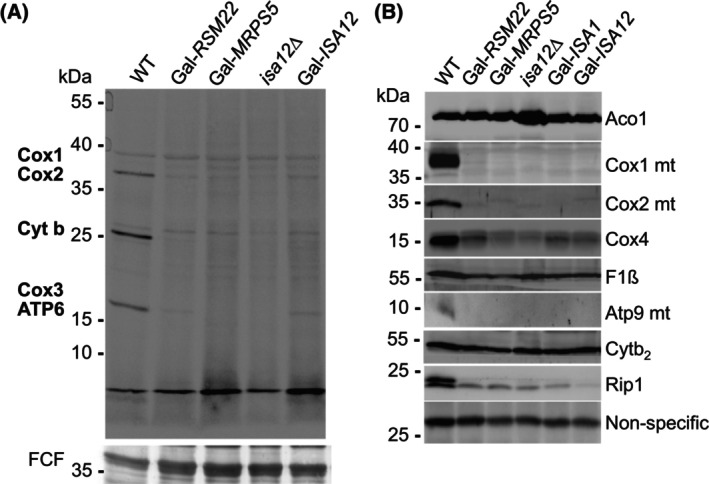
Rsm22, Isa1, and Isa2 are required for mitochondrial translation. (A) Top: Wild‐type (WT), Gal‐*RSM22*, Gal*‐MRPS5*, *isa12*Δ, and Gal‐*ISA12* cells were subjected to ^35^S‐Met/Cys radiolabeling in the presence of the cytosolic protein synthesis inhibitor cycloheximide. Before the radiolabeling, respective proteins were depleted in Gal strains for 40 h in SD minimal medium. Cells were washed and extracts subjected to SDS/PAGE and blotting to a nitrocellulose membrane. A representative autoradiogram of the ^35^S‐labeled translation products is shown. Strain *isa12*Δ is devoid of mtDNA (rho^0^ cells) and thus served as a control for specific labeling of mtDNA‐derived translation products. Bottom: A protein stain with fast green dye (FCF) served as a loading control. (B) The indicated cells were cultivated in YPD medium for 40 h, and mitochondria were isolated. Levels of the indicated proteins (mt, mtDNA‐encoded) were determined by Western blotting. A non‐specific cross‐reactive protein band from Cox1 antibody staining served as the loading control. The apparent molecular masses of marker proteins are indicated in kDa.

We then used the Rsm22‐depleted Gal‐*RSM22* cells to measure mitochondrial translation by *in vivo* radiolabeling with ^35^S‐methionine in the presence of the cytosolic translation inhibitor cycloheximide. In wild‐type cells, three distinct radiolabeled species were detected as *bona fide* mitochondrial translation products, since they were not found in depleted Gal‐*MRPS5* cells or in *isa12*Δ control cells that have lost their mtDNA as a consequence of *ISA1* and *ISA2* gene deletions [45, 60, 61] (Fig. 5A). Based on the size of the translation products, we tentatively assigned them as the subunits Cox1 + Cox2 (COX), cytochrome *b* (complex III), as well as Cox3 (COX) + Atp6 (complex V) [77]. Importantly, these mitochondrial translation products were absent in Gal‐*RSM22* cells after 40 h of Rsm22 depletion in minimal SD medium (Fig. 5A). This result is consistent with the finding that Rsm22 is attached to the mtSSU and performs a putative role in mtSSU assembly, similarly as METTL17 [69, 71]. In line with our hypothesis above, also depleted Gal‐*ISA12* cells were unable to synthesize the mitochondrial gene products, despite the fact that these cells maintain their mtDNA (Fig. 4) [8]. These results demonstrate that both Rsm22 and the late‐acting ISC proteins Isa1–Isa2 are required for the synthesis of mtDNA‐encoded proteins.

To further verify these crucial roles of Rsm22 and Isa1‐Isa2 in mitochondrial translation, we isolated mitochondria from various yeast cells cultivated in rich YPD medium (for protein depletion in Gal cells), and determined the amounts of mtDNA‐encoded proteins by immunostaining. Consistent with the results of the ^35^S‐radiolabeling assay (Fig. 5A), proteins Cox1, Cox2 (subunits of COX), and Atp9 (subunit of complex V) were virtually absent in mitochondria isolated from depleted Gal‐*RSM22* cells as well as from depleted Gal‐*MRPS5*, Gal‐*ISA1*, Gal‐*ISA12* cells, or the rho^0^ control strain *isa12*Δ (Fig. 5B). In contrast, various nucleus‐encoded proteins (aconitase (Aco1), the F_1_‐ATPase subunit (F_1_ß), cytochrome *b*_*2*_ (Cytb_2_), COX subunit Cox4, and Rieske Fe/S protein (Rip1)) were detectable in mitochondria isolated from these strains, even in those lacking mtDNA (Fig. 5B). Compared to the protein levels of wild‐type mitochondria, the amounts of Aco1, F_1_ß, and Cytb_2_ varied only slightly in the different strains. In contrast, the levels of Cox4 and especially of Rip1 (subunit of the cytochrome *bc*_*1*_ complex) were significantly decreased in all mutant strains relative to wild‐type, most likely reflecting the fact that none of these cells was able to assemble functional cytochrome *bc*_*1*_ and COX complexes. Together, these results verify that both Rsm22‐ and late‐acting ISC protein‐deficient cells are incapable of synthesizing mtDNA‐encoded proteins. This finding raised the idea that apo‐Rsm22, in the absence of its Isa1‐Isa2‐synthesized [4Fe‐4S] clusters, is not functional in mtSSU assembly, thereby causing the mitoribosomal translation and respiratory chain defects including a COX deficiency. In turn, this scenario would explain the COX defect in (late‐acting) ISC‐compromised cells.

### Cells depleted of Rsm22 lack complex III and IV activities but retain heme

**Fig. 6:**
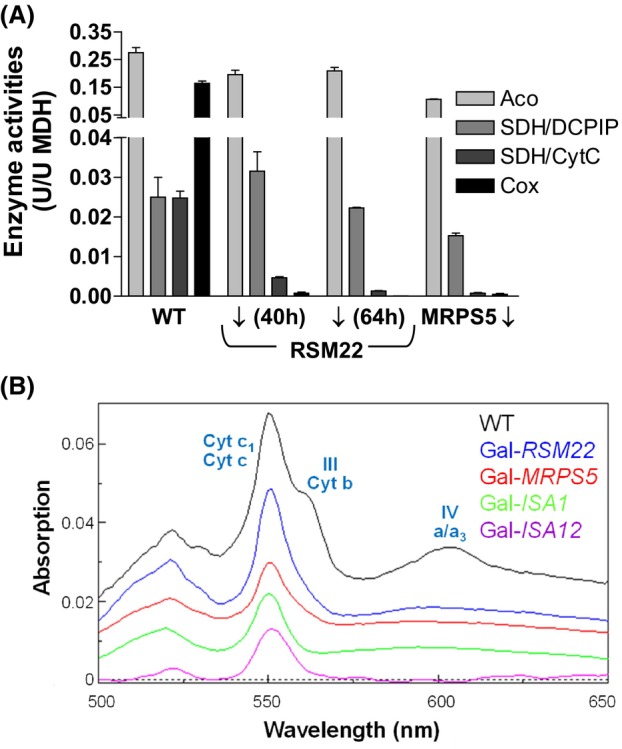
Cells depleted of Rsm22 lack complex III and IV activities but retain heme. (A) Mitochondria were isolated from wild‐type (WT) and depleted (↓) Gal‐*RSM22* or Gal*‐MRPS5* cells. Enzyme activities were measured for respiratory complex II (SDH (DCPIP)), complex II and III (SDH/CytC), and complex IV (COX) relative to the activity of malate dehydrogenase (MDH). Aconitase (Aco) activity served as a control. Error bars indicate the SD (*n* ≥ 4). (B) Reduced‐minus‐oxidized cytochrome spectra were recorded using 1 mg mitochondrial protein from the indicated (depleted) strains. The absorption peaks of the various cytochrome types are indicated. The individual spectra were offset for better visualization.

To experimentally test this model, we measured the enzyme activities of the respiratory complexes III and IV in mitochondria isolated from depleted Gal‐*RSM22* cells or depleted Gal‐*MRPS5* control cells. As a reference, we determined the activities of complex II (SDH) and aconitase (both nucleus‐encoded [4Fe‐4S] enzymes). In support of the model, Gal‐*RSM22* cells lacked COX activity after 40 h or 64 h of Rsm22 depletion, while retaining wild‐type activities of complex II (DCPIP assay) and aconitase (Fig. 6A). Depleted Gal‐*MRPS5* control cells showed a similar phenotype, despite a slightly (30%) lower complex II activity compared to wild‐type. Moreover, both strains displayed strongly decreased activities of respiratory complex III, as assessed by the reduction of cytochrome *c* by succinate, which requires the combined activity of complexes II and III (SDH/CytC assay; Fig. 6A). This defect is readily explained by the lack of mtDNA‐encoded cytochrome *b* synthesis in these cells, rather than an assembly defect of the Rieske [2Fe‐2S] cluster, because this latter reaction occurs independently of the late‐acting ISC proteins. Overall, these findings are consistent with a general block of mitochondrial translation in the absence of Rsm22 or the late‐acting ISC system.

As a final confirmation of the model, the cytochrome spectra of mitochondria isolated from depleted Gal‐*RSM22* and Gal‐*MRPS5* cells were recorded. The spectra lacked the signals corresponding to cytochrome *b* and heme *a/a*_*3*_, while retaining the peak for cytochrome *c/c*_*1*_, albeit at decreased levels compared to wild‐type, likely due to a decrease in cytochrome *c*_*1*_ (Fig. 6B). Importantly, these spectra were strikingly similar to those obtained for depleted Gal‐*ISA1* and Gal‐*ISA12* cells, strongly suggesting that the COX defect in cells with low levels of late‐acting ISC factors was the result of an impaired mitochondrial translation.

**Fig. 7:**
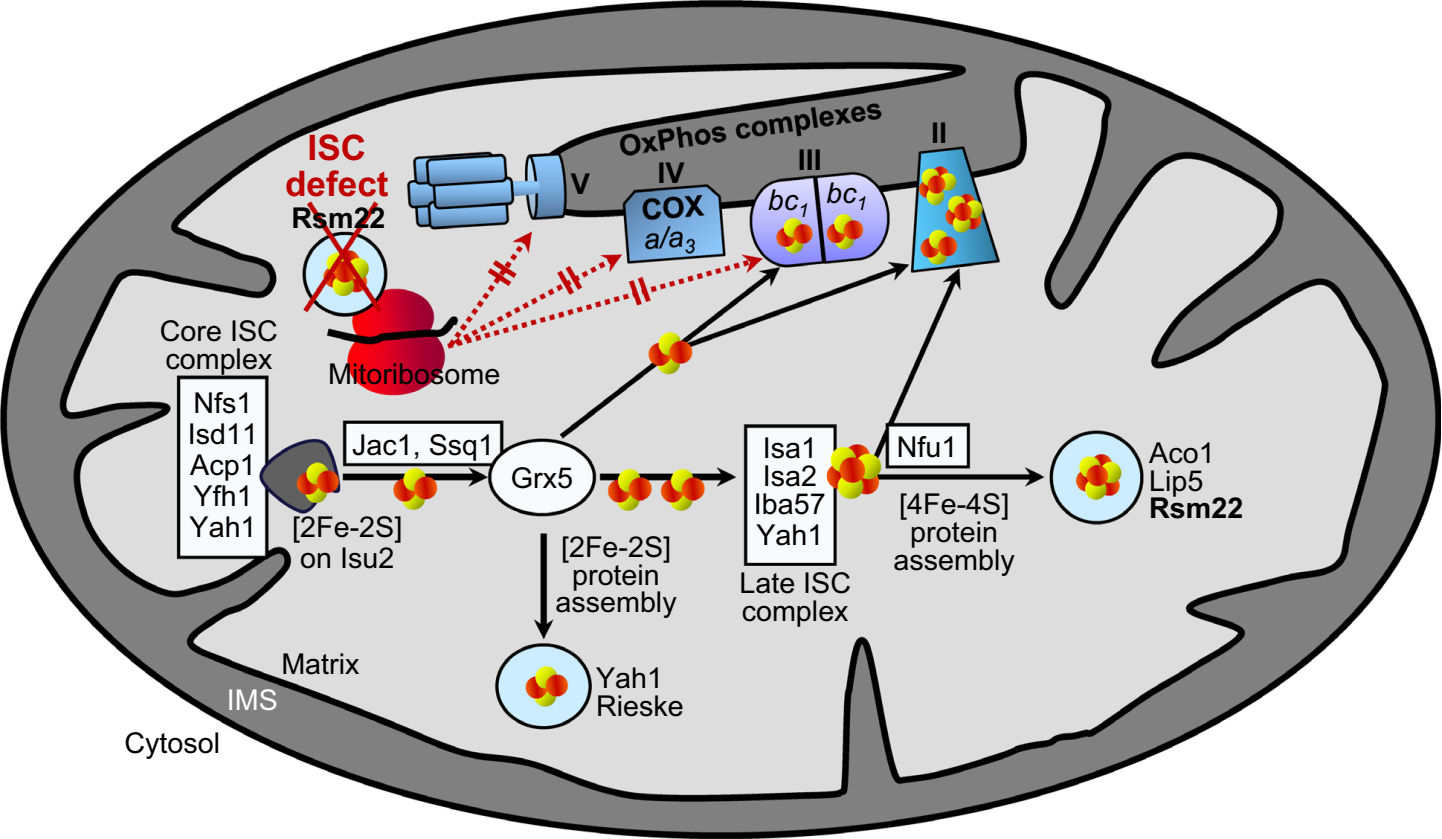
Model for the deficiency of cytochrome oxidase in ISC‐depleted mitochondria. The mitochondrial ISC machinery (yeast nomenclature) synthesizes [2Fe‐2S] and [4Fe‐4S] clusters in several consecutive steps requiring core ISC proteins, transfer proteins, and late‐acting ISC proteins (for details on the ISC system and nomenclature see Introduction and Ref. [5]). Failure of the late‐acting ISC system leads to selective impairment of [4Fe‐4S] protein assembly such as aconitase (Aco1) or respiratory complex II, while [2Fe‐2S] proteins such as Rieske Fe/S protein of complex III are still generated. As shown in this work, the mtSSU assembly protein Rsm22 holds a [4Fe‐4S] cluster *in vivo*, like its mammalian counterpart METTL17 [69, 70]. Defective Fe/S maturation of this protein as a consequence of ISC defects (red cross) leads to impaired maturation of Rsm22, and as a consequence, defective mitoribosomal assembly and protein translation (red dotted arrows). This diminishes all mtDNA‐encoded proteins with the consequence of a respiratory defect because functional OxPhos complexes III–V can no longer be assembled. The model explains why defects in mitochondrial Fe/S cluster assembly indirectly, via Rsm22, lead to a complex IV (COX) defect, even though this enzyme does not contain a Fe/S cluster. Depletion of Rsm22 does not affect complex II (SDH) activity with its three Fe/S clusters because the enzyme contains only nucleus‐encoded proteins. Further details see text. IMS, intermembrane space; red and yellow circles, iron and sulfur ions.

Taken together, our comparative biochemical analyses of various yeast mutant strains strongly indicate that the COX deficiency observed upon ISC protein dysfunction is satisfactorily explained by a defective [4Fe‐4S] cluster maturation of the methyltransferase‐like Rsm22 (Fig. 7). This failure in turn leads to impaired assembly of the mtSSU and defective translation of mtDNA‐encoded OxPhos subunits, thereby preventing the assembly of the respiratory complexes III and IV (i.e., COX).

## Discussion

In this work, we have addressed the longstanding question of why the non‐Fe/S, heme‐containing respiratory complex IV (COX) is severely defective when mitochondrial Fe/S protein biogenesis is compromised, especially the late‐acting ISC system that is specifically responsible for the generation of [4Fe‐4S] proteins. We first show that the COX defect in ISC‐deficient cells occurs with only a slight delay after the loss of Fe/S protein activities, indicating that this phenotype is not a long‐term and mechanistically rather indirect effect, but is tightly connected to the shutdown of the late‐acting ISC system. The COX deficiency was not caused by defective heme synthesis because mitochondrial extracts depleted of late‐acting ISC proteins were capable of synthesizing Zn‐protoporphyrin, demonstrating that these cells contain an active ferrochelatase enzyme. Further, late‐acting ISC‐depleted mitochondria contained *c*‐type cytochromes (part of cytochrome *c* and the *bc*_*1*_ complex III), despite the absence of *b*‐ and *a/a*_*3*_‐type cytochromes (parts of the *bc*_*1*_ complex III and COX, respectively). Notably, the conversion of heme *b* to heme *a* requires the [2Fe‐2S] cluster ferredoxin Yah1 (or human FDX1), but no [4Fe‐4S] proteins [6, 38, 68]. The absence of complex III and IV activities in cells depleted of late‐acting ISC proteins suggested a defect in the translation of mtDNA‐encoded proteins (Fig. 7). We therefore concentrated our further analyses on Rsm22, the yeast homolog of the human mitoribosomal assembly factor METTL17, which recently has been characterized as a [4Fe‐4S] protein [70].

Rsm22‐METTL17 proteins belong to the large family of methyltransferases [78], some of which bind S‐adenosyl‐methionine (SAM) as an essential cofactor in a conserved binding pocket [79]. Controversial findings have been published concerning the methyltransferase enzyme activity of the Rsm22‐METTL17 proteins [70, 73, 79, 80, 81]. At least for mammals, the controversy might be resolved by recent structural work unraveling the role of human METTL17 as an assembly factor for the formation and compaction of the head domain of mtSSU intermediates [69, 82]. Thereafter, METTL17 recruits METTL15, the active 12S rRNA methyltransferase for m4C and m5C methylation [83]. This scenario would explain the methylation defect upon loss of METTL17 as an indirect consequence due to failed METTL15 function. Despite the overall low sequence conservation of Rsm22‐METTL17 relatives, their general function seems to be conserved in eukaryotes, because also Trypanosome and Plasmodium Rsm22 homologs are important for the maintenance of normal mtSSU structure and mitoribosomal translation [84, 85].

In contrast to mammalian METTL17, much less is known about *S. cerevisiae* Rsm22, which initially has been identified as a mtSSU‐associated protein [71]. The *RSM22* gene knockout causes respiratory deficiency [71] and confers tolerance to hypoxic conditions [86], consistent with a mitoribosome assembly function. Interestingly, in some other fungi like *S. pombe*, the gene of Rsm22 is fused to that of the mitochondrial inner membrane protein Cox11, a copper delivery factor for COX maturation [87]. The resulting fusion protein Rsm22‐Cox11 is proteolytically cleaved after import into mitochondria to leave Rsm22 in the matrix space, where it specifically binds to the mtSSU [88]. Like all other members of the eukaryotic METTL17 family, yeast Rsm22 also contains a four Cys motif that is not present in other methyltransferases [73] (Fig. 3A; Fig. S1). Recently, this region in Rsm22/METTL17 has been shown to bind a [4Fe‐4S] cluster required for its stability [69, 70]. Moreover, since overexpression of METTL17 could rescue frataxin‐deficient cells, maturation of this Fe/S protein was proposed to be a rate‐limiting factor for sustaining mitochondrial translation [70].

In our work, we used a ^55^Fe radiolabeling‐immunoprecipitation method [50, 89] to verify that yeast Rsm22 binds a Fe/S cluster *in vivo*. From the dependence of cluster assembly on the late‐acting ISC system, the presence of a [4Fe‐4S] type on Rsm22 can be concluded (Fig. 7). This finding prompted us to analyze whether depletion of the late‐acting ISC system is associated with a mitoribosomal translation defect, as expected from the Fe/S maturation defect of Rsm22 in these cells. Specific ^35^S‐methionine radiolabeling of mtDNA‐dependent gene products showed their complete absence upon depletion of either late‐acting ISC proteins or Rsm22, consistent with the idea that the apoform of Rsm22 was incapable of supporting mtSSU assembly, which prevented mitoribosomal translation. These defects of Rsm22‐depleted cells, in turn, led to a complete deficiency of respiratory complexes III and IV, similar to late‐acting ISC protein deficiency (Fig. 7) [8, 9]. Cytochrome spectra convincingly documented that heme biogenesis is functional in these cells, and a heme deficiency cannot be accounted for these defects. Collectively, the impaired Fe/S cluster maturation of Rsm22 under ISC deficiency may fully explain the associated COX defect (Fig. 7).

Gene deletions for both Rsm22 and the late‐acting ISC proteins are also associated with a mtDNA loss, for so far unclear reasons. This phenotype is frequently observed for many other mitochondrial proteins and is not restricted to factors directly involved in mtDNA maintenance, including replication, transcription, and nucleoid assembly. Mitochondrial protein translation has long been known to be critical for mtDNA maintenance [90]. Molecular reasons for the mtDNA loss include oxidative stress upon defective assembly of the respiratory chain and mitochondrial fusion/fission defects [39, 91]. In the case of Rsm22 and the late‐acting ISC proteins, it seems likely that the impaired assembly of the respiratory complexes and the resulting oxidative stress may be a major driver for the mtDNA loss.

Mammalian METTL17 proteins contain a “LYR” motif that was claimed to be recognized by the mitochondrial ISC protein HSCB (yeast Jac1) to assist in the direct insertion of Fe/S clusters from the core ISC system into target proteins like complex II (SDH; [92]). Mutation of this motif destroys the mtSSU assembly function of METTL17, clearly documenting the importance of these residues [70]. Our study, together with previous observations in yeast and human cells, showed that Rsm22 maturation, like that of all mitochondrial [4Fe‐4S] proteins including SDH, depends on the late‐acting ISC proteins [8, 9]. These findings argue against a direct Jac1‐ or HSCB‐assisted cluster transfer from the core ISC system to Rsm22, bypassing the late‐acting ISC system (Fig. 7). Rather, both Grx5 (human GLRX5) and late‐acting ISC proteins have been shown to be essential for this process [93, 94]. This view is consistent with the observation that the frequently occurring “LYR” consensus motif ([ILV]‐[FY]‐[KR]; [95]) is not fully conserved in Rsm22‐METTL17 proteins (Fig. S1A–C). Particularly in plant family members, the motif is not recognizable at this position, despite the high conservation of this region in plant METTL17 members (Fig. S1C). The essentiality of the “LYR” segment in human METTL17 [70] may therefore be explained by a stabilizing role, together with other conserved short segments (Fig. S1D), for the OB fold domain carrying the forth Cys residue for Fe/S cluster coordination. This rRNA‐binding domain is moderately conserved, even though it shows characteristic differences between different branches of eukaryotic species (Fig. S1A–C).

Collectively, our findings, along with published data (e.g., [69, 70, 71]), suggest the model (Fig. 7) in which the [4Fe‐4S] cluster maturation defect of the methyltransferase‐like Rsm22 in mitochondria with a compromised ISC system explains the concurrent impairment of COX, even though this respiratory complex does not contain a Fe/S cluster. According to the model, the absence of holo‐Rsm22 leads to an assembly defect of the mtSSU. The resulting failure of mtDNA‐encoded mitoribosomal protein synthesis does not allow the assembly of functional OxPhos complexes III‐V, resulting in a respiratory deficiency, similar to cells lacking the (late‐acting) ISC system. A key distinction between Rsm22‐depleted cells and those lacking late‐acting ISC proteins is complex II, which remains fully active in the former but is inactive in the latter cells. This difference is readily explained by the fact that complex II assembly requires three Fe/S clusters and four nucleus‐encoded subunits, but no mitoribosome‐translated proteins. Our results obtained for yeast Rsm22 likely apply to all eukaryotic Rsm22‐METTL17 family members because they all contain the four Fe/S cluster‐binding Cys residues (Fig. 3A; Fig. S1). Interestingly, the recent discovery of three functionally important [2Fe‐2S] clusters in the human mitoribosome adds another layer of explanation why defects in the mammalian (core) ISC system cause a COX failure (Fig. 7) [96, 97, 98]. Less clear is the degree of contribution by the mitochondrial tRNA methylthiotransferase CDK5RAP1, which also binds [4Fe‐4S] clusters. Since CDK5RAP1 knockout cells maintain mitochondrial translation, even though at diminished level [99], this protein defect may have only a minor impact on COX activity. We conclude that the general explanation for the COX defect in ISC‐compromised cells is a Fe/S maturation defect of Rsm22/METTL17.

## Author contributions

UM contributed to conceptualization, data acquisition, data analysis, and writing of the original draft. DT, WS, and LB contributed to data acquisition and analysis. RL contributed to conceptualization, data analysis, funding acquisition, project supervision, and writing of the manuscript. All authors have read and edited the manuscript.

## Peer review

The peer review history for this article is available at https://www.webofscience.com/api/gateway/wos/peer‐review/10.1002/1873‐3468.70129.

## Supporting information


**Fig. S1.** Multisequence alignments and structural segments of eukaryotic members of the Rsm22/METTL17 protein family.
**Fig. S2.** Depletion of mitoribosomal proteins Rsm22 or Mrps5 induces a respiratory deficiency.
**Fig. S3.** Depletion of mitoribosomal proteins Rsm22 or Mrps5 does not cause a loss of mtDNA.
**Table S1.** Yeast strains used in this study.

## Data Availability

All data are contained within the manuscript and the accompanying Supporting information.
