# Supplementary material for: The cytochrome oxidase defect in ISC‐depleted yeast is caused by impaired iron–sulfur cluster maturation of the mitoribosome assembly factor Rsm22
Source: FEBS Lett. 2025 Aug 6;599(16):2301–17. doi: 10.1002/1873-3468.70129 (PMC12375894; doi:10.1002/1873-3468.70129)
Supplement: Supplementary file 1 — Fig. S1. Multisequence alignments and structural segments of eukaryotic members of the Rsm22/METTL17 protein family. Fig. S2. Depletion of mitoribosomal proteins Rsm22 or Mrps5 induces a respiratory deficiency. Fig. S3. Depletion of mitoribosomal proteins Rsm22 or Mrps5 does not cause a loss of mtDNA. Table S1. Yeast strains used in this study. [file FEB2-599-2301-s001.pdf]

Supplementary information for

The cytochrome oxidase defect in ISC-depleted yeast is caused by impaired iron–sulfur cluster maturation of the mitoribosome assembly factor Rsm22

Ulrich Mühlenhoff, Dominik Trauth, Weronika Śliwińska, Linda Boss, Roland Lill

Content:

Supplementary Figures S1 – S3

Supplementary Table S1

Supplementary References

Supplementary Figures

A

Fungal Rsm22

```
            355                 373    379                    400
Sacc.cere   DAELGNISSN YFLKVIAPCP HQRKCPLQVG NPNFYTHKE- GKDLKFCNFQ KSIKRPKFSI ELKKGKLLAT SW----DG-- -----SQGNA SRLKGTGRRN GRDYEILNYS YLIFERSHKD ENTLKEIKKL
Sacc.euba   DVGSMNIPSD YFLKVIAPCP HQRKCPLQVG NPNFYTHKE- GKDLKFCNFQ KSIKRPKFSI ELKKGRLLAT SW----DN-- -----SQGDA SRLKGTGRRN GRDYEILNYS YLILQRSHKD EKSLKEIEKL
Zygo.roux   EKGRGDENNN YHLKIIAPCP HHRKCPLQIG KPQYYEYPE- GKNLKFCNFQ KSITRPKFTM EHKKGKMLAT PWQEPTDG-- -----IGKKG LAKPGTGRPN GRNYEILNYS YLIVERSSTD EQTLQDIETQ
Lach.fant   APSSQDHTGT YHLKVIAPCP HHRKCPLQIG KPQYYEYGK- GKGLKFCTFQ KSVHRPRYAI ELKKGRILAT PWQSKLEP-- -----VGIKG LAREGTGRPN GRNFEILNYS YLVLERSQTD AETVESIIKR
Kluy.marx   ETEEEPNEEN YHIKIIAPCA HHRKCPLQIG KPHYYDLSE- GSKLNFCNFQ KTVLRPKFTI ELKKGKILAT PWQTPTDG-- -----IGIKG KSSAGSGRKN GRGFEIVNYS YLIAERSAKD RETSQSIKAE
Cybe.fabi   TTTDTPAKPD YHISIIAPCS HHKKCPLQTL NPKYYNYPS- GKKLNWCHYQ QSVERPRFLM ELKRGQVLSS KWEKKDEI-- -----SPSRG SG--GSGRVG ANNYEMTNYS YLIAQRSAND AETIDEIETD
Pich.inco   AESFDKLDEQ IDLSIVAPCS HHGKCPLQYF DPHVYLYGQI GKKLKFCSFG VSVQRPKYLL ELKRGKKLAT KWTSTNSG-- -----LAIDG IAKPGRGREN GKDYEVASYS YLIVNRSTED PEVLEKQRQE
Cand.rail   AEDDVNGPID YHLKIIAPCS HHAKCPLQLG NPTFYKIPKY KNKLQFCSYH QTVQRPKFTL ELKRGKKLAT TWDKSSQDGF GFDRMNKRTV QKLEGSGRPG GNDYESGSYS YLIVERSHND TKTLADIDEQ
Cand.albi   NEDSEELEVD YHLSVIAPCP HHRKCPLQLG DPKYYKIPDH RHRLNFCSFS KIVARPPYVI ELKKGKKLAI RWDKSAEDGI G--SISRNAV KRLAGSGRPG GRDTEVGSYS YLIVERSAND LETIKKIEDL
Aspe.nige   SPEEDTFIEK EAGMIVAPCT NHNKCPM--- ---YQIPGHT KGRRDYCHFQ QRYIRPAFLQ RIIGAKDRNH EDLKFSYIA- ----VQRGVD RRIEDSIIQG TEAADAAFDG YEDAVEMPKQ ETEASANEAV
Consensus   .......... yhl.!!APC. hh.KCP$q.g .p.yy..... g..l.fC.%q ..v.RPk%.. elk.gk.lat .w.k...... .......... ....gsgr.g g..y#...ys Yli.ers..# .et...ie..
                               F      F                       F

            473                                                   513
Sacc.cere   RNENVNGKYD IGSLGDDTQN SWPRIINDPV KRKGHVMMDL CAPSGELEKW TVSRSFSKQI YHDARKSKKG DLWASAAKTQ IKGLGDLNV- --KKFHKLEK ERIKQLKKEE RQKARKAMES YNELEDSLQF
Sacc.euba   RNESGNVKYD VGSSGDDTQD TWPRVINDPV KRKGHVMMDL CAPSGELEKW TVSRSFSKQA YHDARKTKKG DLWTLGAKTQ IKGLGDLNV- --KKFKKLEK ERIKQLKKEE RQKSREIIES YNDLEDSLQF
Zygo.roux   R--SAQTTYE LGSLGDNTQS TWPRIIKQPL KRKGHVTMDL CGSSGELEKW IVPKSFGKEA YHDARKASKG DLWGLDAKTK MKSVVNFNV- --AKFQAIEK ELIKQKKREA KRKDREVSEV YNEITHG-EN
Lach.fant   R-ESSNEVYE VGSLGDDTPD TWPRIIKQPL KRKGHVVMDL CGSSGQVEKW TVPKSFSKEA YHDARKALKG DLWALDAKTK APGMGGLNV- --KKLEKVEK DKIREERRQL KKKDREIKEA LNEVDYN-DD
Kluy.marx   R-EENKTKYD IGSLGDNTAN TWPRIIKQPI KRKGHVTLDL CAPSGNFEKW TVPRSLDKNI YHDARKAQKG DLWALEAKTK IQGGGALNV- --KKLEKLHK EEIKKMKNAV KVKSRELKDI ANQLDQEKED
Cybe.fabi   R-ENGSQEFD IGTVTDDV-S LWPRILMSPL KRKGHVTMAL CAASGEVEKW TVTKSFDKVG YHDARKAMAG DLWPLDAKTK MIGKSNQDAG VREKLENYER ELLMKTKKET RLRSKDDRKK FQTKAAAMMN
Pich.inco   LMSGERPTRE IGYKTQER-D EYPRILAPPM KKKGFVIMDL CAPSGHLEKW HISKSVGKQD YHDARKANMG DLWPLGYKSA RQSTKE-NTF YFEKIEEKRE NLRKKKRRDA TKLKRKIAQE YKEAMEA-DP
Cand.rail   R------KFQ NEQLNAADAN NWARIIRQPT KQKQNVKLDV CAPSGRIEIW DVPKSVGIQE YHDARKVERG DLWALGKKTV TVRNIMSDEK V-ERMKIESK MSKKKFLKDE RRK--KWKKI VSSTEGAFDS
Cand.albi   R------SYS DNVIDENDIT NWPRIIATPN KLKRNVKLTV CSTEGNVETW QVPKSLGKQT YHDARKSQLG DSWALAVK-- ---------- --DKLDVLYK TSKKTFKKEQ QRK--RWKKK VGTSVEEFED
Aspe.nige   ASETQPTPTE QQETGGVNFL SLPRIVYQPM KRRGHVIFDL CTPAGKIERW TVPRSFSRQA YKDARKSQWG DLWALGAKTR VARSLRLGE- --KHGEGKKE RLARRAAEKQ EMEEEEEDAL EERMEPTSSM
Consensus   r.e....... .g..g..... .wpRIi.qP. KrkghV..dl CapsG..EkW t!pkSf.kq. YhDARK...G DlWaL.aKt. .......... ...k.e...k .l.k...... ..k....... ..........
                                                  F                    “L YR”         CT
```

OB fold

```
            600                            628
Sacc.cere   DDHQFSNFEV MKKLSTFHGN DFLQHVNRK
Sacc.euba   GNNKLSNIEV MKEMSNVHGH YFSQHVNKK
Zygo.roux   GDVDTLAQVL AHEYQRNRGK KDKKRED
Lach.fant   TTHEQSVDAL AAMYGNEFDR TKK
Kluy.marx   LDPQSAVDAL AKFHQHNFHL ENAKKEKKYA NRKLAVDEY
Cybe.fabi   KGGE-SPEEM VEFYTNKFNM SKRQRQMDKK NGIQQFYD
Pich.inco   EDLEGNLIRM SRIDAYEFLS KPKEEEKMKD KRKFKF
Cand.rail   ENFMTVADEL ATELESSKKY KTEGKRAGFR GDPRDHDGL
Cand.albi   GFFAT--EVM ASQLESSKKY KQQARQAQFE EDPSKFDGN
Aspe.nige   PDIQTPLKKK GQKIPSWEKQ QLKKKRRQAL KQVRPSA
Consensus   .d........ .......... .......... .........
```

(Fig. S1, continued)

B

Vertebrate, fly, worm METTL17

```
           315                  333    339          347                                                                                                404
Homo.sapi  KGKEKSPLDP RPGFVFAPCP HELPCPQLT- ---NLACSFS QAYHPIPFSW NKKP-KE--- --EKFSMVIL ARGSPEEAHR ---WP--RIT QPVLKRPRHV HCHLCCPDGH MQHAVLTARR HGRDLYRCAR
 Bos.taur  NGKEKSPLDP RPGFVFAPCP HELPCPQLT- ASKPLACSFS QAYYPIPFSW NKKP-KE--- --EKFSLVIL ARGSPEKANR ---WP--RIT QPVLKRPRHV HCHLCCPDGH MQHAVITARR HGRDLYRCAR
Ratt.norv  GDKEKSPLDL RPSFVFAPCP HELPCPQLN- AAKSLACSFS QAYQPIPFHW NKKP-KE--- --ETFSMVIL ARGSPKEANR ---WP--RIT QPVLKRPRHV HCHLCCPDGH MQHAVVTARR HGRDLYRCAR
 Mus.musc  GEKEKSPLDL RPSFVFAPCP HELPCPQLN- ASKSLACSFS QAYHPIPFNW NKKP-KE--- --EIFSMVIL ARGSPKEANR ---WP--RIT QPVLKRPRHV HCHLCCPDGH MQHAVVTARR HGRDLYRCAR
Cani.lupu  KGKEKSPLDP RPGFVFAPCP HELPCPQLA- ASKPLACSFS QAYHCIPFSW NKKKQKE--- --EKFSMVIL ARGRPEEAPR ---WP--RLT QPVLKRPRHV HCHLCCPDGH MQHAVITARQ HGRDLYRCAR
Dani.reri  KREDEILHDP RRPSIFAPCT HELKCPKLI- RQPVVPCNFF QFYYSLPLSR SQER-QQ--- --ERFSYLIV SRSDGEKAQK AEHWDMARVI AQVHRRPRHV QCQLCCVNGE LKQLAVTARR HGKDMYRCAR
Dros.mela  EARQFLLEQE QEGHTVAPCP HDLVCPRLRD LADRTPCNFP ISYAPLRLGS DFLADRH--- -TSLYSYAIL KKGSQSDSSS S--WP--RIV RPTLVRSKHA ICRLCTEKGT LQEVIFTKSK HGKSAYSCAK
Dros.sera  EARRFLLEQD DEGHTLAPCP HDVACPRLKD LADRTPCNFP ISYAPLRLGS DSNADRH--- -TSLYSYAIL KKGPQSDSSR S--WP--RIV RPTLVRSKHA ICRVCTGEGN LQEVIFTKSK HGKSAYSCAK
Tele.dalm  DLLLTLGDDN NKCHVLAPCP HDLPCPRLNN LTDRTPCNFE AAYKPLHIG- EVKSERQ--- -IARYSYVIM KKGELQDETR D--WP--RLV RPTIQRSKHV ICRMCTADAN LQEIIFTKSK HGSSVYQCAK
Aede.aegy  FLLKKFESSE VEYHIFSPCP HHQQCPRMK- LDDGTPCNFE VTYNPLPLGK SSE--AS--- -KFLYSYVVF KKGTPTAQSR ---HP--RLV RPTLVRSKHS ICRMCTDGGK LQEIIFTASK HGKNLYRCAR
Brad.copr  HMNEKSESGA VG-HIFAPCA HNMTCPRLL- ADDRSPCNFV TSYMPLPLDP EGANLPQ--- -KELYSYVVL KKGPNTDCDK ---WP--RIV RPTLIRSKHA ICRMCTKDGK LQEVIFTQSK HGKLPYRCAR
Caen.eleg  PPGHTVPTML PTATVLAPCP HDLGCPLGV- ---HSSCTFN TRFQPIRADG RRSEKESDGT EVSKFTYMIL EKSQRKVNNE HT-----ERI LKNRKLGGHV TCDVCTAFRG IQRITLSKK- HGE-MYTALR
Caen.bovi  PSSQAVPTIL PPATVLAPCP HDLGCPMRA- ---KRSCTFS ARYKAIRADG RKSKRESDGT EVTKFTFVIM EKGQRRVG-E QN-----DRI LMNRKLGGHV TCDVCTAFSG IERFTLTKKK HGN-VYSQLR
Hete.trif  PDHVPVPTRL EPGHVFAPCP HDQACPLLSN ---EKSCKFK VRWQEFRTDH RTKSVNRDGT LTGEFSFLIM EKGERKPAEA VP-----ARL LQLERAHKCV SCHICTAFDG LQQLVVSKR- HAK-VYARIR
Aphe.bess  IDGLNIPTAL DVGHVVAPCP HDSGCPLFVG RDYQSSCLHR LKYKETRVDG RSKSQTNSGL AQFSFSYVIL AKGERPPG-I LH-----SRL LTKSTSPACV SCRLCTPNEG LQRFQISKR- AGP-LYSKVR
Consensus  ......p... ...hvfAPCp H#l.CP.l.. ......C.F. ..%.p..... .......... ....%syv!l .kg....... .. wp  r.. .p.l.r..hv .C..Ct..g. l#....tk.. HG...Y.car
                             F      F            F                                                                                         F                  "LYR"
                                                                                                                     OB fold
```

```
           430                        456
Homo.sapi  VSSWGDLLPV LTPSAFPPST -A–QDPSES
 Bos.taur  VSSWGDLLPV ITPLELPPSA -QDAQDAPES
Ratt.norv  VSSWGDLLPV ISPSEFPPPS -PA-EEPPES
 Mus.musc  VSSWGDLLPV IAPSEFPPSS -PD-EEPPEN
Cani.lupu  VSSWGDLLPV ITPSESELPP -SPAEDPLRV DKDA
Dani.reri  NSDWGDLLPV FHKEDEHTIN -DETE
Dros.mela  VSRWGDRLPM SLGQPQIKQE -SIESEEPPV LA
Dros.sera  VSRWGDRLPM SLGQPQVKPG -STELTLATE QADLA
Tele.dalm  VTDWGHRLPI SIGE-KFDTE -TKHTKFLKQ KAEIQKLNQS ETENVREV
Aede.aegy  ASKWGDQLPI KIDNLE
Brad.copr  SSKWGDRLPI SIDAPTEI
Caen.eleg  SRRDGDLLPI NLKTMTSSGI FEVDN
Caen.bovi  SRRDGDVFPA DLKKLTGSGM FNVVLDD
Hete.trif  RAMPSQLLPV NMEVVKSEID LLRNSETLEE QKRQRMLSIS DAAQIESLK
Aphe.bess  SAREGRLFPL EEKIVSSDSD LHDIRQIIDD VEKEYRNKNV E
Consensus  .s.wGdllP. .......... .......... .......... ........
                  CT
```

(Fig. S1, continued)

C

Plant Rsm22

```
            282                300    306       315
Arab.thal   KDGKEVLDLK SGAHIVAPCP HDGKCPLENT GKYCHFVQRL QRTSSQRSYK RTKG-VPLRG FEDEKFCFVA FRRGQRPREL WPLDGMKLET LKERRANKKP EDLEIDYEDF IKSQVVEVPY IDPRAYDSDT
Oryz.sati   SIVCQEASLK NGAFVVAPCP HDGRCPLENT DKYCHFVQRL ERTSSQRAYK RSNG-VPLRG FEDEKFCYVA LRRGKRPEEA WPLDGMKFET LKERHAKRNP EDLIIDYDEQ FPSEEDEEAP VN--AEDC-L
Peri.frut   KTSTDLMTLN TGAFVVAPCP HDGPCPLLNT EKYCHFVQRL ERTSSQRLYK RSKG-SPLRG FEDEKFCYVA FRRGTRPRES WPLDGMEFET LKEMQANRIP ENLEIDVENQ FDSEEYEEDA DNTTSNEL-N
Popu.tric   EPCKDLVPYK GGAFVIAPCS HDGSCPLVKS GKYCHFVQRL QRTTSQRAYK RSKG-ETLRG FEDEKFSFVA FRRGQRPRKP WPLDGMRFET LKEQHAKRNP VDLEIDYEDL IEQNQPEEEV P---YEEVDP
Pyru.comm   EASKDLVTQK SGAFIVAPCS HDGQCPLEKA GKYCHFVQRL ERTSVQRAFK RSKGGQPLRG FEDEKFSFVA FRRGQRPREP WPLDGMKFET LKEQHAKRSP EDLEIDIDEL ISSQQVDL-V P---FEELNP
Bras.rapa   EAGKEVLDLK SGAHIVAPCP HDGKCPLENT AKYCHFVQRL QRTSSQRSYK RTKG-VPLRG FEDEKFCFVV FRRGQRPR-- ---------- ---------- ---------- ---------- ----------
Consensus   e.gk#vldlk sGAh!VAPCp HDGkCPLent .KYCHFVQRL #RTSSQRsYK RtkG vPLRG FEDEKFc%Va fRRGqRPre. wpldgm..et lke..a...p e.l.id.... ..s...e... .........
                               F      F         F

            411                                                   468                                                                            537
Arab.thal   MDENEEEQED GGGTDEDEED KIEEEIEEES ERASVGGGWG RIIFPPFRKG KQVTLDMCVP TKEDGSEGAF ERRVITKSKN PDLHLQAKKS FWGDLWPLTT QQEN--GKKK QVDAEWCRPD EDQKWSGWP
Oryz.sati   VPYDSDAQEL GLFHET---- --EEEFEEQS VRADLGGGWG RIIYSPIRRG RQVQLDVCRA TKRDASEGAF ERVVITQSKN PTMHHQARRS LWGDLWPV
Peri.frut   LSEAADATTG NGNAAE---- --EEGEEEEA PRADLGTGWG RIIYMPFRRG KRVELDICRA TNSEGTEGSF DRVVVTQSRN PKLHHQARRS MWGDLWPLRS GK-N--AKYF M
Popu.tric   VYYDSDVIET DTNDDN---- --DEEEQEEE AHADLGGGWG RIVFSPFKRG RQVTLDVCRS NNRDNSEGSF ERIVVTKSKS PALHYQARRS HWGDLWPF
Pyru.comm   VNYDSDVMEP DVVDEN---- --NEDEEDET GHADLGGGWG RIIYMPVRRG KQVTMDVCRS TKLDGSEGEL QRVVVTKSKN PTLHLQARKS IWGDLWPF
Bras.rapa   ---------- ---------- ---------- GRASVGGGWG RIIFPPFRKG KQVTLDMCVP TNEEGSEGAF ERRVITKSKN PHLHLQAKKS FWGDLWPLTT QQQEITKENK KVDAEWCRPD QDQKWGAWP
Consensus   .......... .......... ...e..ee.. .rAsvGgGWG RII%pPfRkG kqVtLDmCvp Tne#gsEGaF #RrV!TkSkN P.$HlQAkkS fWGDLWPltt qq.......k .vdaewcrpd .dqkw..wp
                                                                  F                         "LYR"            CT
                                                  OB fold
```

(Fig. S1, continued)

D

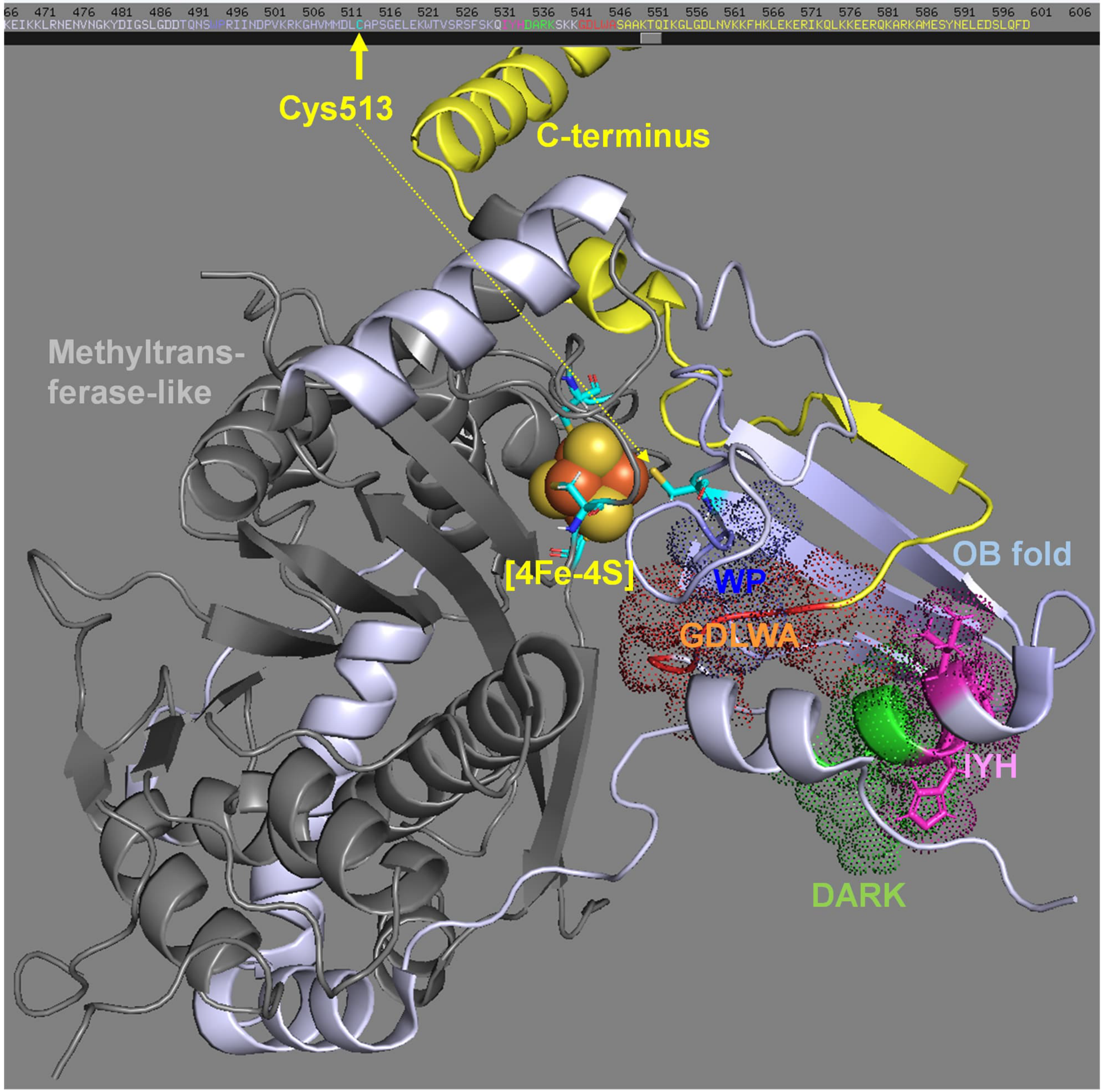

(Fig. S1, continued)

Figure S1: Multi-sequence alignments and structural segments of eukaryotic members of the Rsm22/METTL17 protein family. Alignments were generated by Multalin ([1]) for selected examples of (**A**) fungal Rsm22, (**B**) vertebrate, fly, and worm METTL17, and (**C**) plant Rsm22. Despite the overall low sequence conservation of the Rsm22/METTL17 protein family in eukaryotes, all members have a shared domain structure (Fig. 3A) [2], and contain four conserved Cys residues (highlighted in yellow) which coordinate a [4Fe-4S] cluster (F) in *S. cerevisiae* (Sach.cere) and human (Homo.sapi) members (this work; [3,4]). The Cys motif is unique to this family of methyltransferase-like proteins. The Cys residue numbers are given for the top protein in each alignment. The first three Cys residues are part of the putative SAM-binding methyltransferase-like domain. For simplicity, the N-terminal residues in front of the conserved Cys motif have been deleted in the alignments. The reasonably conserved oligonucleotide-binding (OB) fold (blue bar) contains one of the cluster-binding Cys residues, carries a conserved WP (or WG) dipeptide at its N-terminal side, and is followed by three short segments (purple, green and red) which are uniquely conserved in the presented three eukaryotic species families. (**D**) These sequence parts tightly interact in the 3D structures of Rsm22/METTL17 and coordinate the [4Fe-4S] cluster via Cys513. The “LYR” motif suggested to bind to the HSPB chaperone is not fully conserved, and completely missing in plant METTL17 examples (C). Part **D** provides a 3D rendition (Pymol; [5]) of the structural domains and segments of *S. cerevisiae* Rsm22 (PDB ID: 8OM2; [3]) with the color codes for the various indicated segments according to parts A-C.

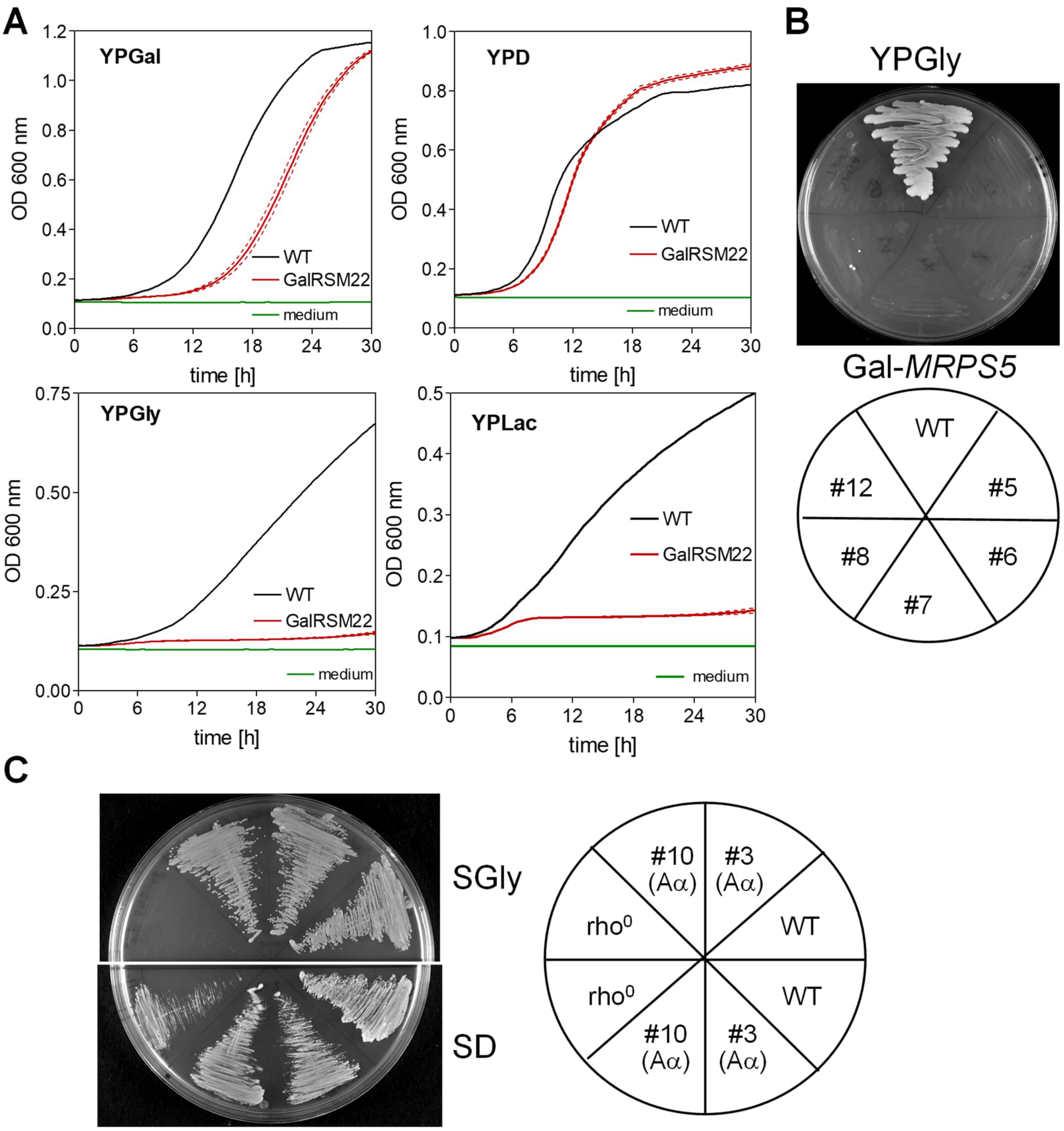

Figure S2: Depletion of mitoribosomal proteins Rsm22 or Mrps5 induces a respiratory deficiency. (A) Wild-type (WT) and the isogenic Gal-*RSM22* cells (W303 strain background) were grown in the indicated liquid media. Gal-*RSM22* failed to grow in the presence of the non-fermentable carbon sources lactate (Lac) and glycerol (Gly). Gal-*RSM22* cells were depleted of Rsm22 by cultivation in YPD medium for 40 h prior to analysis. Low levels of Rsm22 explain the lag phase on SGal medium which requires active mitochondrial respiration for proper growth. **(B)** WT cells and several independent Gal-*MRPS5* clones were grown on YPGly medium. As expected from the mitoribosomal defect upon depletion of Mrps5, depleted Gal-*MRPS5* cells failed to grow indicating a respiratory defect, similar to depleted Gal-*RSM22* cells in part A. **(C)** In order to assess the maintenance of mtDNA, Gal-*RSM22* cells were depleted for 40 h in YPD medium, and then mated

with a matα rho^0 tester strain (BY4742 background; *mrs3::HIS3; mrs4::LEU2*; *trp1::kan*MX4). The resulting diploid cells were cultivated on solid minimal SC medium supplemented with glucose (SD) or glycerol (SGly). Diploid cells generated with Gal-*RSM22* strains (two independent clones are shown) grew on the non-fermentable carbon source glycerol, indirectly indicating the maintenance of mtDNA in the depleted Gal-*RSM22* cells.

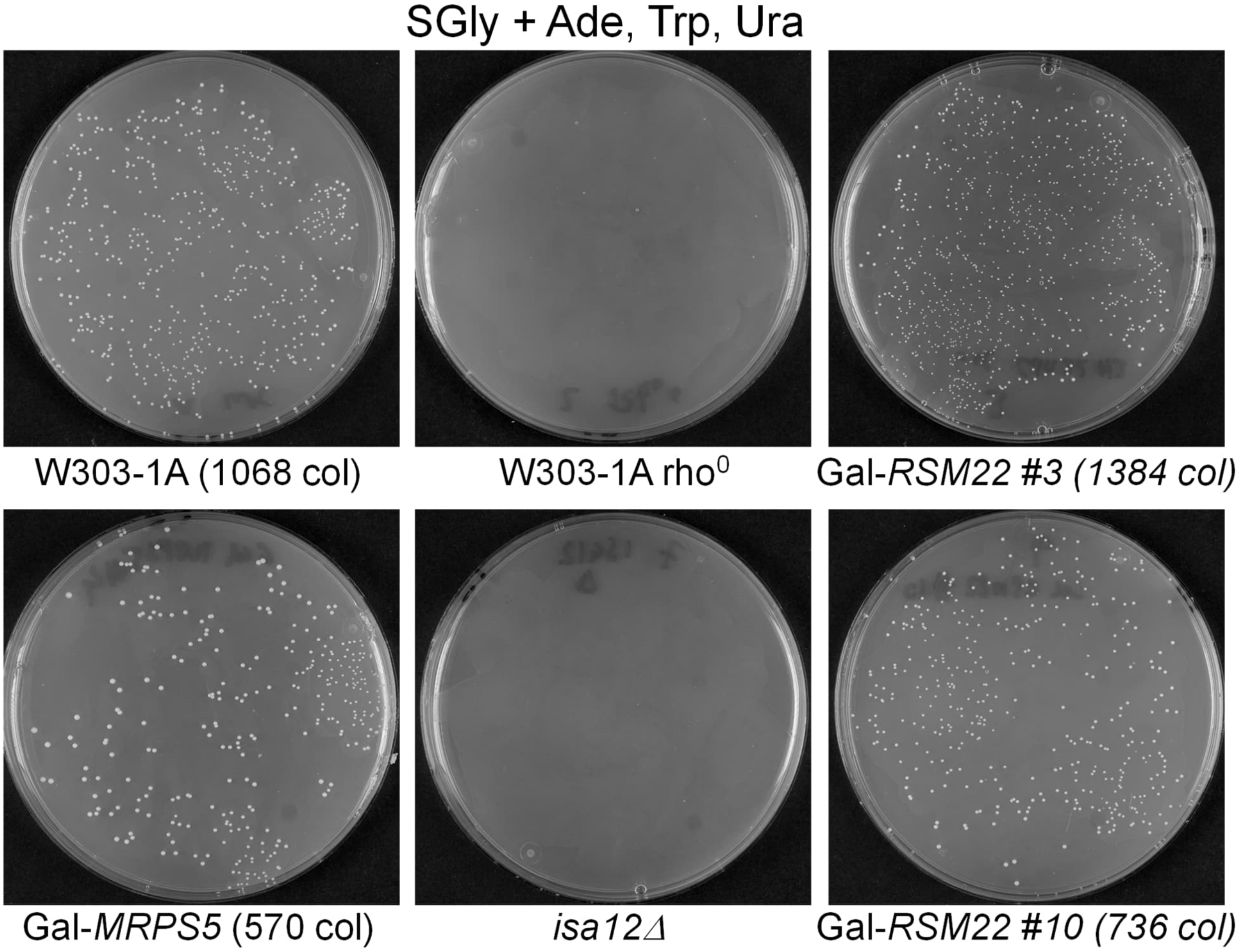

Figure S3: Depletion of mitoribosomal proteins Rsm22 or Mrps5 does not cause a loss of mtDNA. To semi-quantitatively assess the presence of mtDNA in yeast, Gal-*RSM22* and Gal-*MRPS5* cells were depleted for 40 h. These cells, wild-type W303-1A cells, and the negative rho^0 control cells W303-1A and *isa12*Δ (W303-1A background) were subjected to mating by mixing with the same amount of matα rho^0 tester strain (BY4742 background; *mrs3::HIS3; mrs4::LEU2*; *trp1::kan*MX4). Cells were sedimented, resuspended in 500 µl YPD, and incubated for 4.5 h at 30°C. Cells were sedimented, washed twice with water and 50% were cultivated on agar plates with minimal SC medium supplemented with glycerol (SGly) and the indicated supplements for 4 days at 30°C. Since this medium lacked leucine and histidine (required for growth of W303-1A, but not for the BY4742 tester strain), as well as lysine (required for growth of BY4742, but not W303-1A), only diploid cells with intact mtDNA were able to grow. For quantitative analysis, the number of colonies (col) were counted. Due to the lack of selectable markers, the Gal-*ISA12* strain was not included in the analysis.

Supplementary Table

Table S1: Yeast Strains Used in This Study

Strain	Genotype	Method of Generation	Source (Reference)
W303-1A	*MATa; ura3-1; ade2-1; trp1-1; his3-11,15; leu2-3,112*		[6]
Gal-*ISA1*	*pISA1::GAL1-10-HIS3*	PCR fragment (pFA6a-HIS3-Gal) [7]	[8]
Gal-*ISA12* (Gal-*ISA1*/Gal-*ISA2*)	Gal-*ISA1; pISA2::GAL1-10-LEU2*	PCR fragment (pUG73)[9]	This work
Gal-*IBA57*	*pIBA57:: GAL-L-natNT2*	PCR fragment (pYM-N27) [10]	[11]
Gal-*RSM22*	*pRSM22:: GAL-L-natNT2*	PCR fragment (pYM-N27) [10]	This work
Gal-*MRPS5*	*pMRPS5:: GAL-L-natNT2*	PCR fragment (pYM-N27) [10]	This work
Gal-*HEM15*	*pHEM15:: GAL-L-natNT2*	PCR fragment (pYM-N27) [10]	This work
Gal-*NFS1*	*pNFS1::GAL1-10-HIS3*	PCR fragment (pFA6a-HIS3-Gal) [7]	[7]
Gal-*SSQ1*	*pSSQ1::(GAL1-10)-HIS3*	PCR fragment (pFA6a-HIS3-Gal) [7]	[7]
Gal-*YAH1*	*pYAH1::GAL1-10-HIS3*	PCR fragment (pFA6a-HIS3-Gal) [7]	[7]
Gal-*YFH1*	*pYFH1::GAL1-10-HIS3*	PCR fragment (pFA6a-HIS3-Gal) [7]	[7]
*isa12*Δ	*isa1::kanMX2; isa2::HIS3*	PCR Fragment (pFA6a-kanMX2; pFA6a-HIS3MX6 [12]	[13]
*cyt2*Δ	*cyt2::LEU2*	gene replacement	[14]
*rip1*Δ	*rip1::LEU2*	PCR fragment (pUG73)[9]	This work
rho^0 tester strain	*BY4742* (MATα) *mrs3::HIS3, mrs4:LEU2*	Ethidium bromide treatment	[15]

All strains were created in W303-1A background. *GAL*-promoter exchange strains were depleted by cultivation in rich YPD medium for 40 h except for Gal-*ISA1*, Gal-*SSQ1* and Gal-*YFH1* which were depleted for 64 h. Cultures were diluted to OD_{600nm} = 0.1 after 16 h and, where necessary, after 40 h.

Supplementary References

[1] Corpet F. (1988). Multiple sequence alignment with hierarchical clustering. *Nucleic Acids Res* **16**, 10881-10890.

[2] Alam J, Rahman FT, Sah-Teli SK, Venkatesan R, Koski MK, Autio KJ, Hiltunen JK and Kastaniotis AJ. (2021). Expression and analysis of the SAM-dependent RNA methyltransferase Rsm22 from Saccharomyces cerevisiae. *Acta Crystallogr D Struct Biol* **77**, 840-853, doi:10.1107/S2059798321004149.

[3] Ast T, Itoh Y, Sadre S, Mccoy JG, Namkoong G, Wengrod JC, Chicherin I, Joshi PR, Kamenski P, Suess DLM *et al.* (2024). METTL17 is an Fe-S cluster checkpoint for mitochondrial translation. *Mol Cell* **84**, doi:10.1016/j.molcel.2023.12.016.

[4] Harper NJ, Burnside C and Klinge S. (2023). Principles of mitoribosomal small subunit assembly in eukaryotes. *Nature* **614**, 175-181, doi:10.1038/s41586-022-05621-0.

[5] DeLano WL. (2002) The PyMOL Molecular Graphics System (http://www.pymol.org).ed.^eds)

[6] Mortimer RK and Johnston JR. (1986). Genealogy of principal strains of the yeast genetic stock center. *Genetics* **113**, 35-43.

[7] Mühlenhoff U, Gerber J, Richhardt N and Lill R. (2003). Components involved in assembly and dislocation of iron-sulfur clusters on the scaffold protein Isu1p. *EMBO J* **22**, 4815-4825.

[8] Mühlenhoff U, Gerl MJ, Flauger B, Pirner HM, Balser S, Richhardt N, Lill R and Stolz J. (2007). The ISC proteins Isa1 and Isa2 are required for the function but not for the *de novo* synthesis of the Fe/S clusters of biotin synthase in *Saccharomyces cerevisiae*. *Eukaryot Cell* **6**, 495-504.

[9] Gueldener U, Heinisch J, Koehler GJ, Voss D and Hegemann JH. (2002). A second set of loxP marker cassettes for Cre-mediated multiple gene knockouts in budding yeast. *Nucleic Acids Res* **30**, e23.

[10] Janke C, Magiera MM, Rathfelder N, Taxis C, Reber S, Maekawa H, Moreno-Borchart A, Doenges G, Schwob E, Schiebel E *et al.* (2004). A versatile toolbox for PCR-based tagging of yeast genes: new fluorescent proteins, more markers and promoter substitution cassettes. *Yeast* **21**, 947-962.

[11] Gelling C, Dawes IW, Richhardt N, Lill R and Mühlenhoff U. (2008). Mitochondrial Iba57p is required for Fe/S cluster formation on aconitase and activation of radical SAM enzymes. *Mol Cell Biol* **28**, 1851-1861.

[12] Wach A, Brachat A, Poehlmann R and Phillipsen P. (1994). New heterologous modules for classical or PCR-based gene disruptions in *Saccharomyces cerevisiae. Yeast* **10**, 1793-1808.

[13] Pelzer W, Mühlenhoff U, Diekert K, Siegmund K, Kispal G and Lill R. (2000). Mitochondrial Isa2p plays a crucial role in the maturation of cellular iron-sulfur proteins. *FEBS Lett* **476**, 134-139.

[14] Steiner H, Zollner A, Haid A, Neupert W and Lill R. (1995). Biogenesis of mitochondrial heme lyases in yeast. Import and folding in the intermembrane space. *J Biol Chem* **270**, 22842-22849.

[15] Mühlenhoff U, Stadler JA, Richhardt N, Seubert A, Eickhorst T, Schweyen RJ, Lill R and Wiesenberger G. (2003). A specific role of the yeast mitochondrial carriers MRS3/4p in mitochondrial iron acquisition under iron-limiting conditions. *J Biol Chem* **278**, 40612-20, doi:10.1074/jbc.M307847200.
